# Healthcare Resource Utilization and Discharge Readiness in Adult Hospitalized Patients With Candidemia or Invasive Candidiasis Who Received an Echinocandin: An Analysis of United States Hospitals

**DOI:** 10.1093/ofid/ofad703

**Published:** 2024-01-03

**Authors:** Thomas P Lodise, Kevin W Garey, Jalal A Aram, Brian H Nathanson

**Affiliations:** Department of Pharmacy Practice, Albany College of Pharmacy and Health Sciences, Albany, New York, USA; Department of Pharmacy Practice, University of Houston College of Pharmacy, Houston, Texas, USA; Melinta Therapeutics, Parsippany, New Jersey, USA; OptiStatim, LLC, Longmeadow, Massachusetts, USA

**Keywords:** candidemia, echinocandin, invasive candidiasis, outcomes

## Abstract

**Background:**

Scant real-world outcomes data are available among hospitalized patients with candidemia (C) or invasive candidiasis without candidemia (IC) who were treated with an echinocandin and few have assessed if there is an opportunity to accelerate the transition of their care to the outpatient setting. This study described the outcomes associated with echinocandin therapy for C/IC and determined the proportion of patients on an echinocandin at hospital discharge (HD) who were potentially eligible for an earlier HD.

**Methods:**

A retrospective, multicenter observational study was performed using the PINC AI Healthcare Database (January 2016–April 2019) of hospitalized adult patients with C/IC who received ≥3 days of an echinocandin. Outcomes included post–index culture hospital costs and discharge location. Patients were considered potentially dischargeable earlier than actual HD day if they met the following 3 criteria prior to their actual HD day: resided on a non–intensive care unit hospital ward until HD, received any oral medications, and had no diagnostic/therapeutic interventions.

**Results:**

A total of 1865 patients met study criteria. Mean (standard deviation) post–index culture hospital costs for patients with C and IC were 50 196 (64 630) US dollars and 61 551 (73 080) US dollars, respectively. Of the 1008 patients on an echinocandin near HD and discharged alive, 432 (42.9%) were potentially dischargeable prior to their actual hospital day. Most patients (35.8%) were discharged to a long-term care facility.

**Conclusions:**

The findings suggest that a high proportion of hospitalized C/IC patients receiving an echinocandin near the time of HD were potentially dischargeable earlier. Like all studies of this nature, the findings need to be prospectively validated.

## Teaser

Hospital costs of patients with invasive candidiasis who received an echinocandin were substantial and most required additional medical care postdischarge. Many patients (43%) on an echinocandin at hospital discharge were potentially dischargeable prior to their actual discharge day.

Candidemia (C) and invasive candidiasis (IC) are 2 of the most common healthcare-associated infections in the United States (US) [1, 2] and are associated with significant morbidity, mortality, and healthcare costs [3–8]. Current practice guidelines recommend echinocandins as first-line therapies for C/IC due to their superior response rates, association with improved survival, robust microbiologic activity against azole-resistant *Candida* spp, favorable safety profiles, and limited drug–drug interactions [9, 10]. However, there are scant real-world healthcare resource utilization (HRU) data available among adult hospitalized patients with C/IC who received definitive treatment with an echinocandin. Additionally, it is well documented that many patients with invasive infections require additional medical care in another healthcare facility or with a home health agency postdischarge, but limited information is available on the hospital discharge destinations of hospitalized patients with C/IC who were treated with an echinocandin [11].

It is also unclear if there is an opportunity to accelerate the transition of care of adult hospitalized patients with C/IC who received definitive treatment with an echinocandin to the outpatient setting to reduce C/IC-associated healthcare costs and the risks associated with prolonged hospitalizations. While continued hospitalization is often required for unstable patients with comorbidities or those with more severe infections, studies across multiple infectious diseases demonstrate that most clinically stable patients with modest diagnostic, therapeutic, and monitoring requirements can be safely managed as outpatients [12–15]. A recent study demonstrated that one-third of all echinocandin courses in hospitalized patients were continued until the last day of hospitalization, and one-half of patients discharged from the hospital continued echinocandin therapy [11]. Additionally, a recent observational cohort study from 20 European countries that assessed data from 632 patients with C reported that hospital stay was extended specifically for the purpose of completing parenteral antifungal treatment in 100 of 621 (16%) patients by a median of 14 (interquartile range [IQR], 3–23) days [16]. These findings [11, 16] suggest that many patients with C/IC receiving echinocandins complete most of their treatment course in the hospital, regardless of their clinical status and medical care requirements. However, there are a lack of data across US hospitals on the percentage of patients who received an echinocandin at the time of hospital discharge and were potentially eligible for an earlier hospital discharge.

The objective of this study was to describe the HRU outcomes of hospitalized adult patients with C/IC who received definitive treatment with an echinocandin in a large database of hospitals in the US. As hospital reimbursement and antimicrobial stewardship programs are both increasingly tied to quality, efficiency, and cost of care [17–19], we also sought to determine the proportion of patients on an echinocandin at time of hospital discharge who were potentially eligible for an earlier hospital discharge relative to their actual hospital discharge day.

## METHODS

### Study Design and Population

We conducted a retrospective, multicenter observational study among adult hospitalized patients with IC who received treatment with an echinocandin between 2016 and 2019 in the PINC AI Healthcare Database, formerly known as the Premier Healthcare Database [20]. The PINC AI Healthcare Database is a large, US hospital-based, service-level, all-payer database that contains information on inpatient discharges, primarily from geographically diverse nonprofit, nongovernmental, and community and teaching hospitals and health systems from rural and urban areas, representing approximately 25% of annual US inpatient admissions. Because this study utilized already existing Health Insurance Portability and Accountability Act (HIPAA)–compliant fully de-identified data, it was exempt from institutional review board review [20].

Patients from the PINC AI Healthcare Database were included in this study if they met the following criteria: (1) age ≥18 years; (2) hospital discharge between January 2016 and December 2019; (3) presence of a *Candida* sp from a designated culture site that is consistent with C or IC; and (4) receipt of ≥3 days of an echinocandin starting within 2 days prior to or after index C/IC culture collection day. The first qualifying C/IC diagnosis during the study period was defined as the index admission. For patients with ≥1 C/IC admission with echinocandin treatment during the study period, only the first admission was analyzed. If a patient had >1 positive C/IC culture within 1 hospitalization, the first C/IC culture collection day was used to define the index day. Echinocandins received 2 days prior to index day to 2 days after the index day (−2 to +2 days of index day) were classified as empiric. If the index echinocandin treatment day was 3–6 days after index day, it was classified as the early targeted therapy. Treatments received 7–10 days after index day were classified as late targeted therapy, and echinocandin treatment first administered >10 days after index day was classified as delayed therapy.

### Baseline Data Covariates

Hospital-level covariates included region, hospital size, teaching status, and population served. Patient-level covariates collected during the qualifying C/IC hospital admission included information on demographics, medical history, comorbidities, hospitalization course, microbiology and infection characteristics, diagnostic and therapeutic interventions, antimicrobials (ie, antibiotics and antifungals) and other medications received. Patient demographics included sex, race, age, ethnicity, primary payer, and admission source. Medical history and comorbid conditions included hospitalization ≤6 months of index admission, Charlson Comorbidity Index (CCI) (overall score and individual conditions) [21], and Elixhauser comorbidities [22]. Data collected during hospital course included hospital length of stay (LOS) prior to the index C/IC culture collection day, residence in an intensive care unit (ICU) on the index day, and receipt of mechanical ventilation (MV) on the index day. Microbiology and infection characteristics included all positive clinical *Candida* sp cultures at designed culture site(s) ±3 days of index day. The *Candida* sp, infection type (C/IC), and hospital day of index day in relation to hospital admission day were documented.

Diagnostic and therapeutic procedures included charges categorized as diagnostic imaging, diagnostic services, therapeutic services, endoscopy, neurodiagnostics, nuclear medicine, radiation therapy, and surgery by the PINC AI Healthcare Database. Antimicrobial data included all antifungals/antimicrobials received between the date of admission and index day. The first day of echinocandin treatment in relation to index day and duration of echinocandin therapy were documented. Antifungal therapies administered concurrently with the echinocandin and those administered after discontinuation of echinocandin therapy were quantified. Other medications received on or before the index day included vasopressors, inotropes, diuretics, β-blockers, calcium blockers, and nutritional support.

### Outcomes

Clinical outcomes assessed in the study included hospital LOS from index day to hospital discharge and discharge destination (in-hospital mortality vs hospice vs home vs home healthcare care vs acute-care hospital vs long-term care facility). HRU outcomes assessed included mean hospital costs (overall and ICU) from index day to hospital discharge and 30-day hospital readmissions (all-cause and C/IC-related) among survivors. For hospital costs, total mean costs and average daily costs were determined and costs in ICU and non-ICU were captured separately. Total mean costs from index day to hospital discharge of all pharmacotherapies, all other medical care (defined as all healthcare services and procedures other than pharmacotherapies), and room and board, respectively, were also documented.

The proportion of patients on an echinocandin at the time of hospital discharge who were potentially eligible for an earlier hospital discharge was assessed among study patients who were discharged alive and were receiving an echinocandin near hospital discharge (−2 day of hospital discharge day to hospital discharge day). Based on data from the ICan Discharge study [11], hospital survivors receiving an echinocandin near hospital discharge were considered potentially dischargeable prior to their actual hospital discharge day if they met the following 3 criteria during their hospital course prior to hospital discharge day and then maintained all 3 criteria until hospital discharge: (1) resided on a non-ICU hospital ward; (2) received any oral medications, and (3) did not receive any diagnostic/therapeutic interventions other than insertion of a peripherally inserted central catheter (PICC).

### Statistical Methods

Bivariate analyses were performed to compare clinical and HRU outcomes between patients with C versus IC in the full study population. Wilcoxon or *t* test were used to test for the differences in continuous variables, and χ^2^ tests were used to test for the differences in the distribution of dichotomous or categorical variables unless a cell count was <5, wherein the Fisher exact test was used. Stratified analyses were performed to compare hospital costs between patients with C or IC by (1) presence in the ICU versus non-ICU on index day; (2) hospital survivors versus nonsurvivors; and (3) timing of echinocandin therapy in relation to index day. We used a least absolute shrinkage and selection operator algorithm to identify demographics, comorbidities, and microbiologic, infection, and treatment characteristics that were most strongly associated with costs from index day to discharge. We then used a generalized linear model with a logarithmic link function and gamma distribution to derive adjusted mean (marginal) costs from index day to discharge with 95% confidence intervals for each predictor.

The proportion of patients on an echinocandin at the time of hospital discharge who were potentially eligible for an earlier hospital discharge was quantified and expressed as a percentage among patients in the study who were discharged alive and received an echinocandin near hospital discharge (−2 day of hospital discharge day to hospital discharge day). Additionally, the percentages of patients who may have been discharged from the hospital earlier by 0, 1, 2, 3, and 4 days were determined. The mean (standard deviation [SD]) difference in hospital days between the first potentially eligible hospital discharge day and the actual hospital discharge day was determined among patients who were potentially eligible for an earlier hospital discharge 1 to 4 days prior to the actual hospital discharge day and continued to meet the hospital discharge criteria until hospital discharge. Stratified analyses were also carried out to estimate the proportion of patients on an echinocandin 2 days prior to hospital discharge who were potentially eligible for an earlier hospital discharge prior to their actual hospital discharge day, based on CCI score, C versus IC, and *Candida* sp. For all analyses, *P* values <.05 were considered statistically significant. All analyses were done using Stata/MP 17.0 for Windows (StataCorp LLC, College Station, Texas).

## RESULTS

During the study period, 1865 patients met the study criteria (Supplementary Table 1). Of the 4340 adult hospitalized patients with a *Candida* sp on a clinical culture consistent with C/IC, 2563 received at least 1 day of an echinocandin on or after index day. Of these 2563 patients, 1865 received ≥3 days of echinocandins for their index C/IC during the study period. Among the 1865 patients in the study, 626 had IC and 1239 had C. There were 864 cultures sites among the 626 IC patients, and the culture site listings are shown in Supplementary Table 2. Table 1 compares baseline characteristics and clinical covariates in patients with C versus IC. Baseline characteristics, patient acuity indicators, microbiologic characteristics, and antimicrobial treatment patterns were largely similar in C/IC patients with a few exceptions. Within 2 days of the index blood culture, 70% of C patients received an echinocandin, whereas only 50% of IC patients received an echinocandin.

**Table 1.** Comparison of Baseline Characteristics Between Patients With Candidemia Versus Invasive Candidiasis

Baseline Characteristics	Invasive Candidiasis (n = 626)	Candidemia (n = 1239)
Census region		
Midwest	157 (25.08)	284 (22.92)
Northeast	74 (11.82)	93 (7.51)
South	395 (63.10)	859 (69.33)
West	0 (0.00)	3 (0.24)
No. of beds		
<300	83 (13.26)	245 (19.77)
300–499	166 (26.52)	349 (28.17)
≥500	377 (60.22)	645 (52.06)
Teaching vs nonteaching hospital	440 (70.29)	770 (62.15)
Urban vs rural location of hospital	563 (89.94)	1126 (90.88)
Age, y, median (SD)	60.2 (14.6)	58.3 (16.6)
Sex, female	311 (49.68)	590 (47.62)
Race		
White	505 (80.67)	925 (74.66)
Black	71 (11.34)	195 (15.74)
Asian	7 (1.12)	6 (0.48)
Other	27 (4.31)	82 (6.62)
Unknown	16 (2.56)	311 (25.10)
Hispanic ethnicity	24 (3.83)	42 (3.39)
Admission source		
Nonhealthcare facility (including home)	426 (68.05)	826 (66.67)
Clinic	43 (6.87)	78 (6.30)
Transfer from another hospital	140 (22.36)	271 (21.87)
Transfer from skilled nursing facility or intermediate care facility	8 (1.28)	36 (2.91)
Other	9 (1.44)	28 (2.26)
Payer		
Medicare	334 (53.35)	674 (54.40)
Medicaid	94 (15.02)	263 (21.23)
Managed care	120 (19.17)	167 (13.48)
Commercial	34 (5.43)	39 (3.15)
Other	44 (7.03)	96 (7.75)
Hospitalization in 6 mo prior to index admission	261 (41.69)	566 (45.68)
Any chronic kidney disease diagnosis at admission	123 (19.65)	250 (20.18)
Elixhauser comorbidities		
Congestive heart failure	156 (24.92)	385 (31.07)
Cardiac arrhythmia	257 (41.05)	494 (39.87)
Valvular disease	64 (10.22)	192 (15.50)
Pulmonary circulation disease	74 (11.82)	166 (13.40)
Peripheral vascular disease	78 (12.46)	174 (14.04)
Paralysis	20 (3.19)	61 (4.92)
Other neurological disorders	147 (23.48)	453 (36.56)
Chronic pulmonary disease	176 (28.12)	379 (30.59)
Diabetes without chronic complications	63 (10.06)	116 (9.36)
Diabetes with chronic complications	159 (25.40)	387 (31.23)
Hypothyroidism	96 (15.34)	176 (14.21)
Renal failure	191 (30.51)	392 (31.64)
Liver disease	121 (19.33)	236 (19.05)
Peptic ulcer disease with bleeding	13 (2.08)	26 (2.10)
AIDS	3 (0.48)	14 (1.13)
Lymphoma	10 (1.60)	26 (2.10)
Metastatic cancer	46 (7.35)	97 (7.83)
Solid tumor without metastasis	103 (16.45)	148 (11.95)
Rheumatoid arthritis/collagen vascular	30 (4.79)	66 (5.33)
Coagulopathy	153 (24.44)	375 (30.27)
Obesity	138 (22.04)	256 (20.66)
Weight loss	275 (43.93)	524 (42.29)
Fluid and electrolyte disorders	461 (73.64)	935 (75.46)
Chronic blood loss anemia	22 (3.51)	24 (1.94)
Deficiency anemia	70 (11.18)	131 (10.57)
Alcohol abuse	73 (11.66)	113 (9.12)
Drug abuse	56 (8.95)	230 (18.56)
Psychosis	11 (1.76)	38 (3.07)
Depression	143 (22.84)	299 (24.13)
Hypertension	413 (65.97)	793 (64.0)
Charlson comorbidities		
Acute myocardial infarction	72 (11.50)	149 (12.03)
Congestive heart failure	156 (24.92)	385 (31.07)
Peripheral vascular disease	78 (12.46)	174 (14.04)
Cerebrovascular disease	37 (5.91)	139 (11.22)
Dementia	20 (3.19)	79 (6.38)
Chronic obstructive pulmonary disease	176 (28.12)	379 (30.59)
Rheumatoid disease	25 (3.99)	57 (4.60)
Peptic ulcer disease	77 (12.30)	81 (6.54)
Mild liver disease	54 (8.63)	99 (7.99)
Diabetes	108 (17.25)	228 (18.40)
Diabetes with complications	114 (18.21)	275 (22.20)
Hemiplegia or paraplegia	20 (3.19)	61 (4.92)
Renal disease	191 (30.51)	392 (31.64)
Cancer	78 (12.46)	117 (9.44)
Moderate/severe liver disease	46 (7.35)	67 (5.41)
Metastatic cancer	46 (7.35)	97 (7.83)
AIDS	3 (0.48)	14 (1.13)
Charlson comorbidity score		
0	104 (16.61)	152 (12.27)
1	84 (13.42)	183 (14.77)
2	91 (14.54)	195 (15.74)
3	86 (13.74)	168 (13.56)
4	70 (11.18)	142 (11.46)
≥5	191 (30.51)	399 (32.20)
Mean (SD)	3.3 (2.6)	3.5 (2.7)
Length of stay prior to index culture, days		
Mean (SD)	7.8 (9.8)	7.7 (10.9)
Median (IQR)	5 (2–10)	3 (1–11)
Residence in ICU on index culture day	275 (43.93)	241 (19.45)
MV on index culture day	181 (28.91)	349 (28.17)
Infection type		
Candidemia only	0 (0.00)	1202 (97.01)
Invasive candidiasis only	626 (100.0)	0 (0.00)
Candidemia and invasive candidiasis	0 (0.00)	37 (2.99)
*Candida* sp ±3 d of index culture		
*C albicans*	285 (45.53)	414 (33.41)
*C glabrata*	183 (29.23)	348 (28.09)
*C parapsilosis*	39 (6.23)	176 (14.21)
*C tropicalis*	60 (9.58)	130 (10.49)
Other	140 (22.36)	213 (17.19)
No. of *Candida* sp ±3 d of index culture day		
1	547 (87.38)	1200 (96.85)
2	77 (12.30)	36 (2.91)
≥3	2 (0.32)	3 (0.24)
Antifungals received between admission and index culture day		
Fluconazole	165 (26.36)	156 (12.59)
Voriconazole	1 (0.16)	5 (0.40)
Posaconazole	0 (0.00)	5 (0.40)
Isavuconazole	0 (0.00)	6 (0.48)
Anidulafungin	1 (0.16)	7 (0.56)
Micafungin	109 (17.41)	119 (9.60)
Caspofungin	10 (1.60)	30 (2.42)
No. of antifungals received between admission and index culture day		
0	368 (58.79)	940 (75.87)
1	230 (36.74)	270 (21.79)
2	28 (4.47)	29 (2.34)
Antibiotics received between admission and index culture day		
Aminoglycosides	48 (7.67)	88 (7.10)
β-lactam	601 (96.01)	1103 (89.02)
Fluoroquinolone	101 (16.13)	194 (15.66)
Vancomycin	366 (58.47)	820 (66.18)
Daptomycin	24 (3.83)	65 (5.25)
Macrolide	55 (8.79)	117 (9.44)
Oxazolidinones	37 (5.91)	92 (7.43)
Polymyxins	4 (0.64)	10 (0.81)
Rifamycin	0 (0.00)	1 (0.08)
Sulfa	10 (1.60)	30 (2.42)
Tetracycline	19 (3.04)	69 (5.57)
Other (fosfomycin, nitrofurantoin)	3 (0.48)	3 (0.24)
No. of antibiotics received between admission and index treatment day		
0	8 (1.28)	79 (6.38)
1	182 (29.07)	26 (2.10)
2	276 (44.09)	518 (41.81)
≥3	160 (25.56)	381 (30.75)
EC received for index culture day		
Caspofungin	125 (20.0)	289 (23.3)
Micafungin	500 (79.9)	902 (72.8)
Anidulafungin	4 (0.6)	57 (4.6)
EC treatment initiation relative to index culture day		
Empiric (−2 to 2 d of C/IC)	310 (49.52)	867 (69.98)
Early targeted (3–6 d post-C/IC)	231 (36.90)	342 (27.60)
Late targeted (7–10 d post-C/IC)	49 (7.83)	22 (1.78)
Delayed (≥11 d post-C/IC)	36 (5.75)	8 (0.65)
Duration of EC treatment, days		
Mean (SD)	10.0 (9.0)	8.9 (7.4)
Median (IQR)	7 (4–13)	6 (4–12)
Other antifungals received from index EC treatment day through discharge		
Fluconazole	302 (48.24)	614 (49.56)
Voriconazole	26 (4.15)	45 (3.63)
Posaconazole	1 (0.16)	3 (0.24)
Antifungal therapies administered concurrently with the EC treatment		
Fluconazole	212 (33.87)	340 (27.44)
Voriconazole	16 (2.56)	27 (2.18)
Posaconazole	1 (0.16)	3 (0.24)
Antifungal therapies administered post-discontinuation of EC therapy		
Fluconazole	177 (28.27)	428 (34.54)
Voriconazole	16 (2.56)	30 (2.42)
Posaconazole	0 (0.00)	2 (0.16)
Additional therapies administered on or before in the index culture day		
ACE inhibitors	41 (6.55)	72 (5.81)
β-blockers	285 (45.53)	419 (33.82)
Calcium blockers	128 (20.45)	202 (16.30)
Enteral nutrition	11 (1.76)	12 (0.97)
Total parenteral nutrition	62 (9.90)	79 (6.38)
Vasopressors	326 (52.08)	521 (42.05)
Diuretics	207 (33.07)	360 (29.06)
Inotropes	20 (3.19)	57 (4.60)
Pain medication	606 (96.81)	1042 (84.10)
Sedatives	123 (19.65)	245 (19.77)
Discharge location		
Home	99 (15.81)	249 (20.10)
Home health organization	157 (25.08)	213 (17.19)
Acute-care facility	24 (3.83)	54 (4.36)
Long-term care facility	238 (38.02)	429 (34.62)
Hospice	42 (6.71)	94 (7.59)
Died	66 (10.54)	200 (16.14)
Readmission within 30 d among survivors		
All-cause 30-d readmission	89 (15.89)	135 (12.99)
Readmission for C/IC based on *ICD-10* or DRG codes (DRG = 853–855, 870–872)	20 (3.57)	41 (3.95)

Data are presented as No. (%) unless otherwise indicated.

Abbreviations: ACE, angiotensin-converting enzyme; C/IC, candidemia/invasive candidiasis without candidemia; DRG, diagnosis-related group; EC, echinocandin; *ICD-10*, *International Classification of Diseases, Tenth Revision*; ICU, intensive care unit; IQR, interquartile range; MV, mechanical ventilation; SD, standard deviation.

**Figure 1.**
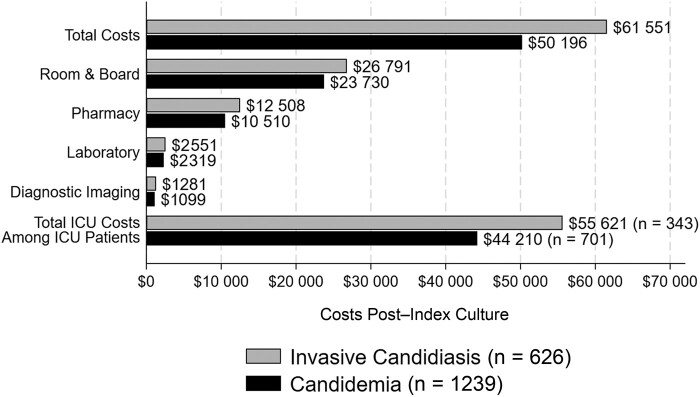
Comparison of mean healthcare cost post–index culture day between patients with candidemia and invasive candidiasis without candidemia (C/IC) overall. Total intensive care unit (ICU) costs among ICU patients reflected total mean costs in the ICU from index C/IC culture day to discharge among patients who received care in the ICU at any point during their hospitalization. Costs are presented as United States dollars ($).

The mean hospital LOS was 16.1 (SD, 17.0) days for patients with C while the mean hospital LOS post–index day for patients with IC was 18.7 (SD, 18.5) days. Mean hospital costs (all costs are US dollars [$]) post–index day exceeded $50 000 in both groups, with higher mean post–index day hospital costs observed in patients with IC ($61 551 [SD, $73 080]) compared to those with C ($50 196 [SD, $64 630]) (*P* < .001; Figure 1). The primary drivers of hospital costs post–index day for both C and IC patients were room and board, followed by pharmacy, laboratory, and diagnostics costs. The mean cost per day in an ICU ward post–index day was $5263 (SD, $4861) while the mean cost per day in a non-ICU ward was $1879 (SD, $1126).

Mean costs post–index culture among patients in the ICU at time of index culture were $86 483 (SD, $91 931) for IC patients and $66 386 (SD, $80 563) for C patients. Mean costs among patients not in the ICU at time of index culture were $42 019 (SD, $45 275) for IC patients and $36 535 (SD, $41 943) for C patients. Mean hospital costs post–index C/IC culture day were significantly higher among nonsurvivors ($83 159 [SD, $111 997]) versus survivors ($49 158 [SD, $55 555]) (*P* < .001). Among C patients, costs post–index culture day among survivors and nonsurvivors were $44 661 (SD, $48 184) and $78 946 (SD, $112 555), respectively (*P* < .001). Among IC patients, mean costs post–index culture day among survivors and nonsurvivors were $57 500 (SD, $66 367) and $95 923 (SD, $110 148), respectively (*P* < .001). For patients with C or IC, mean post–index culture hospital costs were higher among patients who received late targeted or delayed echinocandin treatment relative to those who received empiric or early targeted echinocandin treatment (Supplementary Figure 1). In the regression analysis (Supplementary Table 3), notable factors associated with increased costs post-index culture included timing of echinocandin treatment in relation to index day, receiving MV on the index day, and receiving either fluconazole, total parenteral nutrition, vasopressor, and/or inotropes on or before the index day. Select chronic conditions associated with greater costs include chronic kidney disease, presence of cardiac arrhythmia, pulmonary circulation disorders, coagulopathy, depression, and weight loss. Advanced age (≥70 years) and prior hospitalization within 6 months of the index admission were associated with lower mean costs.

**Figure 2.**
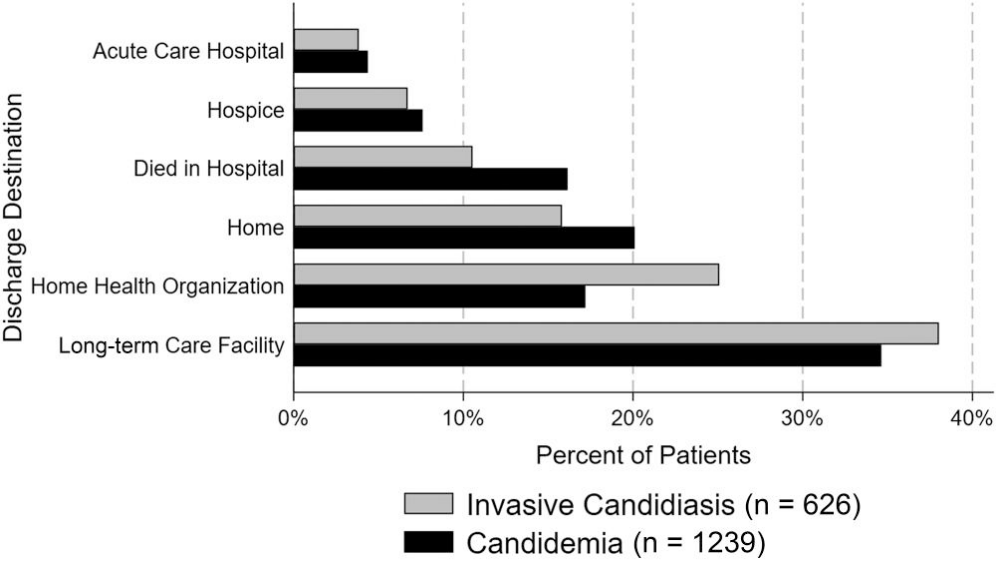
Comparison of discharge destination between patients with candidemia and invasive candidiasis without candidemia.

Hospital discharge disposition of the 1865 patients in the study population by C versus IC are shown in Figure 2 and Table 1. Of the 1239 patients with C, 16% died during their hospitalization, 20% were discharged home, 35% were discharged to a long-term care facility, 4% were transferred to another acute-care facility, 17% were discharged to a home health organization, and 8% were discharged to hospice. Of the 626 patients with IC, 11% died during their hospitalization, 16% were discharged home, 38% were discharged to a long-term care facility, 4% were transferred to another acute-care facility, 25% were discharged to a home health organization, and 7% were discharged to hospice. Of the patients discharged alive, 14% had a 30-day all-cause readmission and 4% had a 30-day C/IC-related readmission.

Sixty-three percent of the hospital survivors (1008/1599) were receiving an echinocandin within 2 days of hospital discharge (Supplementary Table 4). Of the 1008 patients on an echinocandin within 2 days of hospital discharge, 317 had *Candida albicans* on index culture and 30.0% (95/317) of *C albicans*–infected patients received fluconazole on their last hospitalization day. Fifteen percent of patients on an echinocandin within 2 days of hospital discharge (151/1008) had a PICC line inserted within 2 days of hospital discharge and 19% of all hospital survivors receiving an echinocandin within 2 days of hospital discharge had a charge code for a PICC line (ie, supply, maintenance, insertion). The mean and median costs associated with insertion and maintenance of a PICC line were $1114 (SD, $934) and $968 (IQR, $438–$1530), respectively.

**Figure 3.**
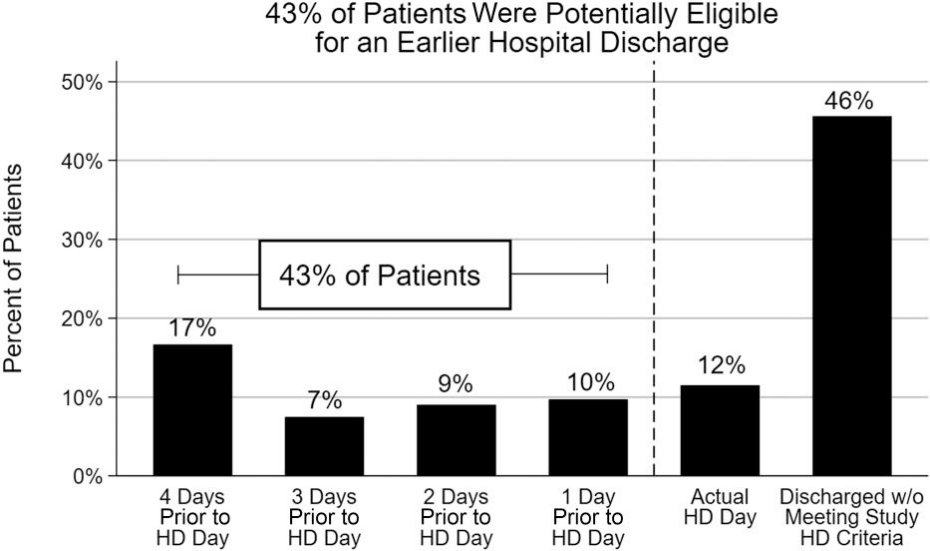
Cumulative percentage of patients on an echinocandin ≤2 days of hospital discharge (HD) who were potentially eligible for an earlier HD. Number of days a patient was dischargeable prior to actual HD day: first day on echinocandin from HD in which the patient resided on a non–intensive care unit ward, received any oral medications, and had no further diagnostic/therapeutic interventions (other than insertion of a peripherally inserted central catheter line). The sum of the percentages do not equal 100% due to rounding.

**Figure 4.**
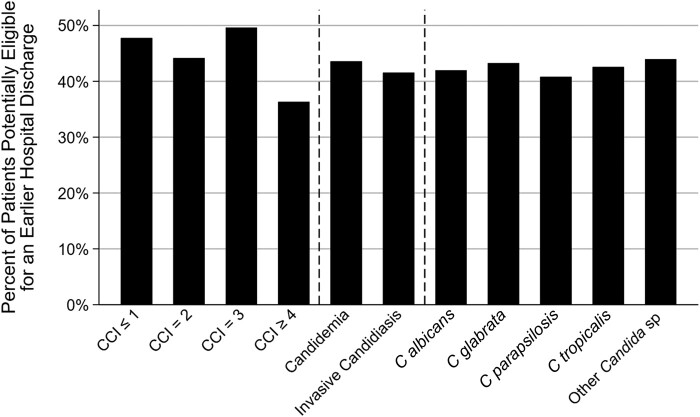
Proportion of patients on an echinocandin ≤2 days of hospital discharge who were potentially eligible for an earlier hospital discharge by Charlson Comorbidity Index score, candidemia and invasive candidiasis without candidemia, and *Candida* sp.

Among the 1008 patients who were discharged alive and were receiving an echinocandin within 2 days of hospital discharge, 42.9% (n = 432) were potentially eligible for hospital discharge prior to actual hospital discharge day (Figure 3). The percentages of patients who were potentially eligible for an earlier hospital discharge by 1, 2, 3, and 4 days were 16.7%, 7.4%, 9.0%, and 9.7%, respectively. Among the 42.9% of patients who were potentially eligible for an earlier hospital discharge, the mean difference in days between satisfying the potential hospital discharge eligibility criteria and actual hospital discharge day was 2.7 (SD, 1.2) days. The proportion of patients who were potentially eligible for an earlier hospital discharge prior to their actual hospital discharge day did not vary substantially by CCI score, C/IC, or *Candida* sp, though those with CCI scores >=4 had the fewest patients potentially eligible for earlier discharge (Figure 4).

Of the 576 patients who were found not to be potentially eligible for an earlier hospital discharge, 116 met all 3 potentially hospital dischargeable criteria on their actual hospital discharge day (ie, last hospital day) while 460 did not meet ≥1 criteria on their actual hospital discharge day. Of the 460 patients who did not meet the proposed hospital discharge eligibility criteria on their actual hospital discharge day, 366 (79.6%) did not meet 1 criterion, 89 (19.3%) did not meet 2 criteria, and 5 (1.1%) did not meet all 3 criteria. Most patients (410/460 [89.1%]) who were discharged without meeting ≥1 of the proposed hospital discharge eligibility criteria had a diagnostic/therapeutic intervention on their hospital discharge day. We also observed that 24.1% (111/460) were discharged from the hospital directly from the ICU and 8.3% (38/460) were not taking oral drugs on their last hospital day/discharge day.

Baseline characteristics, patient acuity indicators, and treatment patterns were largely similar between C/IC patients who met versus did not meet the earlier hospital discharge criteria prior to their actual hospital discharge day with a few exceptions (Supplementary Table 5). Patients who met the proposed hospital discharge criteria earlier than their actual discharge day were more likely to have Medicaid compared to patients who did not meet the earlier hospital discharge criteria. Additionally, the patients who met the proposed hospital discharge criteria earlier than their actual discharge day were less likely to have congestive heart failure, renal disease, and moderate/severe liver disease, had a lower CCI score, and were less likely to be in the ICU or receive MV on index C/IC culture day. We also observed that patients who were potentially eligible for an earlier hospital discharge were more likely to receive another antifungal during their hospitalization.

## DISCUSSION

There were several notable clinical and HRU outcome findings in this retrospective, multicenter observational study. Hospital LOS and costs post–index C/IC culture day were substantial, with higher costs in patients with IC versus C and among patients who were in the ICU on their index culture day. Consistent with other infection burden of illness studies [7, 23, 24], room and board costs represented the largest proportion of total post–index culture hospital costs. Interestingly, the mean costs post–index C/IC culture day were higher among hospital nonsurvivors relative to hospital survivors and were lower among patients ≥70 years of age versus <70 years of age. Timing of treatment in relation to index C/IC culture day was found to be a critical determinant of hospital costs post–index culture for patients with either C or IC; higher hospital costs were observed among C/IC patients who received delayed echinocandin treatment initiation relative to those who received empiric/early targeted echinocandin therapy.

To place the post–index C/IC culture hospital cost findings in better context, the hospital costs associated with C/IC in this study were several-fold higher than those observed in studies of adult, hospitalized patients with infections due to carbapenem-resistant Enterobacterales (CRE), methicillin-resistant *Staphylococcus aureus* (MRSA), or other antibiotic-resistant gram-negative pathogens considered to be urgent or serious threats by the US Centers for Disease Control and Prevention [25–29]. In a recent study of hospitalized adult patients with CRE infections using the same healthcare database, the mean hospital costs post–index culture day were found to be $19 816, with higher hospital costs observed in patients who received delayed versus early appropriate therapy [29]. In a study of adult patients with MRSA sterile-site infections, the median hospital costs in those treated appropriately were $13 688 compared with $19 427 among those who received delayed appropriate therapy [26]. Similarly, mean post–index culture hospital costs among hospitalized adult patients with CRE, carbapenem-resistant *Pseudomonas* sp, or extended-spectrum β-lactamase–producing Enterobacterales were reported to be $21 010 in patients who received timely appropriate therapy and $32 518 in patients who received delayed appropriate therapy. The mean hospital costs post–index C/IC culture observed in this study best align with the post–index culture hospital costs of patients with hospital- or ventilator-associated bacterial pneumonia [30].

The most noteworthy finding from the hospital discharge destination analyses was the high proportion of patients who received additional medical care postdischarge. Among the 1599 hospital survivors, 42% were discharged to a long-term care facility, 5% were transferred to another acute-care inpatient facility, and 23% were discharged to a home under the care of a home health organization. Due to the nature of the PINC AI Healthcare Database, limited information was available on outpatient treatments received and we could not ascertain if patients continued echinocandin treatment or received an oral antifungal (eg, fluconazole, voriconazole) postdischarge. Notably, 31.4% (317/1008) of hospital survivors who were on an echinocandin within 2 days of hospital discharge had *C albicans* on index culture, and 95 *C albicans*–infected patients (30.0%) received fluconazole on the last hospitalization day. This finding suggests that some of these patients may have continued oral fluconazole in the outpatient setting. Additionally, 15% of patients on an echinocandin within 2 days of hospital discharge had a PICC line inserted within 2 days of hospital discharge and 19% of all hospital survivors who recieved an echinocandin within 2 days of hospital discharge had a charge code for a PICC line (ie, supply, maintenance, insertion), suggesting that many patients likely continue echinocandin therapy postdischarge. A recent study showed that almost half of C/IC patients on an echinocandin at hospital discharge received outpatient echinocandin therapy [11], highlighting the need for new treatment options to mitigate the costs and outcomes associated with daily receipt of echinocandins for patients with C/IC in the outpatient setting. Of note, the in-hospital mortality rates for patients with C/IC in this study were slightly lower than observed in other similar studies [5–8, 11]. Seven percent of patients were discharged to hospice, suggesting that the observed in-hospital mortality rates in this study may not reflect the true extent of deaths potentially attributable to C/IC. Furthermore, all patients were required to receive an echinocandin for ≥3 days post–index culture as part of this study, and this may have potentially biased the observed in-hospital mortality rates as some of the patients excluded in this study due to lack of receipt of an echinocandin for ≥3 days likely died.

The results also suggest that a sizeable proportion of hospitalized C/IC patients receiving an echinocandin near the time of hospital discharge had modest diagnostic/therapeutic requirements prior to their actual hospital discharge day and were potentially eligible for an earlier hospital discharge. Of the 1008 patients on an echinocandin near hospital discharge, 42.9% were potentially dischargeable 1 to ≥4 days prior to their actual hospital day. Notably, 33.1% of patients were potentially eligible for an earlier hospital discharge ≥2 days prior to their actual hospital discharge day. The proportion of patients who were potentially eligible for hospital discharge ≥2 days prior to their actual hospital discharge day did not vary substantially by CCI, infection type (ie, C vs IC), or *Candida* sp. These results have important implications for clinical practice as they suggest that many C/IC patients who are receiving an echinocandin at time of hospital discharge in US hospitals are potentially eligible for an earlier hospital discharge. Given the high costs of C/IC management [3–8], the findings highlight the critical need for US healthcare systems to develop well-defined criteria for hospital discharge of clinically stable C/IC echinocandin-treated patients with modest diagnostic, therapeutic, and monitoring requirements as a potential cost-containment measure. However, due to the retrospective observational design of this study, the findings need to be prospectively validated prior to implementation of any hospital policy or pathway that facilitates the discharge of clinically stable C/IC echinocandin-treated patients.

Several factors should be considered when interpreting the study findings from the early hospital discharge readiness analyses. The intent of this analysis was simply to highlight the proportion of adult hospitalized C/IC patients receiving echinocandin treatment who potentially could have been managed in the outpatient setting prior to the actual hospital discharge day. As with other electronic healthcare databases, physical examination findings, imagining and diagnostic test results, and clinical notes were not available and clinical laboratory and vital signs data were limited in the PINC AI Healthcare Database. Instead, we relied on microbiologic culture and treatment data to define patients with C/IC and patient service-level data (eg, diagnostic tests performed, therapeutic services received, medications administered, acuity of hospital care provided [ICU vs non-ICU]) to assess the potential for an earlier hospital discharge. The lack of electronic medical record information prevented us from determining if there were clinical, socioeconomic, or postdischarge placement factors for continued hospitalization in patients who were identified as being potentially eligible for an earlier hospital discharge. Interestingly, patients who met the proposed hospital discharge criteria earlier than their actual discharge day were more likely to have Medicaid. Data show that patients with Medicaid have less access to outpatient care [31] and this may have contributed to the potentially longer hospital stays in patients with Medicaid, but further study is needed to determine the definitive reasons for this observation. It is also possible that C/IC potentially worsened the patients’ other comorbid conditions, and management of their uncontrolled comorbidities may have required them to stay in the hospital to complete their entire course of echinocandin therapy. However, patients who met the proposed hospital discharge criteria earlier than their actual discharge day had a lower disease severity (ie, lower proportion of select comorbidities and less likely to be in the ICU or receive MV on index C/IC culture day), suggesting that the presence of uncontrolled comorbidities was an unlikely explanation for observed findings. Even if our approach missed the presence of unidentified reasons for continued hospitalization in some patients, the study findings would still indicate that a sizeable proportion of adult hospitalized patients with C/IC were potentially eligible for an earlier discharge. Additionally, our findings are consistent with a recent study by Hoenigl et al, which found that hospital LOS was extended only to complete parenteral antifungal treatment in 16% of patients [16]. Given that hospital costs were primarily reflective of room and board costs [32] and many patients only had modest diagnostic, therapeutic, and monitoring requirements at the end of their hospitalization, we believe a conservative application of our findings suggests there is an opportunity to improve the efficiency of healthcare delivery for C/IC patients receiving echinocandin therapy, for example, by shifting inpatient care to the outpatient setting in appropriate patients.

Several other limitations also should be noted. This was a retrospective, observational study and is subject to all the limitations associated with this study design. While the PINC AI Healthcare Database is large and contains coding and billing information for about 50 million admissions from approximately 500 US acute-care hospitals (>350 with microbiologic data), it may not be generalizable to all other US hospitals [20]. As with all electronic health databases, patient, microbiologic, and treatment data were extracted from an electronic database and the potential for inaccuracies exists. However, PINC AI has several validation processes in place to ensure the accuracy of the data [20]. Definitions of study covariates are based on the elements in hospital claims derived from the uniform billing form (UB-04) and categorized into PINC AI standard definitions, as well as PINC AI's proprietary data dictionary [20]. This was a study of hospitalized adult patients with incident (ie, first episode) C/IC who received definitive treatment with an echinocandin. We required all patients to receive an echinocandin for ≥3 days post–index culture and most patients received, on average, 9 days of echinocandin therapy. We selected this restricted population to maximize internal validity and minimize potential biases introduced by shorter durations of echinocandin treatment. As such, it is unknown if the observed findings are applicable to other populations that were not included in the study, including pediatric patients, patients with non-index C/IC episodes, patients who received an echinocandin for <3 days, or those who received definitive treatment with other antifungal therapies. Additionally, post-discharge data were largely unavailable in this study and the projected HRU outcomes associated with C/IC in this study are only applicable to index C/IC events in the hospital. This limitation affects our results in 2 possible ways. First, we may have underestimated deaths post-discharge attributable to C/IC. Second, we may have underestimated the total HRU associated with C/IC as data suggest many patients continue to receive care for their C/IC post-discharge [11]. Last, we did not differentiate C/IC-related outcomes by the echinocandin received; most patients received either micafungin (75.2%) or caspofungin (22.2%). Comparison of outcomes between echinocandin received was outside the scope of this study and merits evaluation in a future study. As such, the C/IC-related outcomes observed in this study may vary by the specific echinocandin available at a given institution.

In conclusion, hospital costs associated with C/IC in this multicenter, retrospective observational study were found to be substantial, with room and board costs representing the largest component. More than 60% of adult hospitalized C/IC patients discharged alive were on an echinocandin near time of hospital discharge. Of the patients on an echinocandin near hospital discharge, 43% were potentially dischargeable prior to their actual hospital day, irrespective of CCI, infection type or *Candida* sp. Most patients who survived their hospitalization required additional medical care in a long-term care facility, in another acute-care facility, or with a home health agency post-discharge. Collectively, the findings from this study highlight the critical need for new treatment approaches and strategies that expeditiously and safely transition appropriate C/IC patients receiving echinocandins to the outpatient setting as a measure to improve the quality and efficiency of their care and curb C/IC-related hospital costs. Like all studies of this nature, the findings need to be prospectively validated to determine the actual proportion of C/IC patients receiving an echinocandin at hospital who were dischargeable prior to their actual hospital day.

## Supplementary Material

ofad703_Supplementary_Data

## Supplementary Data

Supplementary materials are available at *Open Forum Infectious Diseases* online. Consisting of data provided by the authors to benefit the reader, the posted materials are not copyedited and are the sole responsibility of the authors, so questions or comments should be addressed to the corresponding author.

## Notes

***Financial support*.** This work was supported by Cidara Therapeutics, Inc (San Diego, California) and Melinta Therapeutics LLC (Parsippany, New Jersey).
