# Supplementary material for: Healthcare Resource Utilization and Discharge Readiness in Adult Hospitalized Patients With Candidemia or Invasive Candidiasis Who Received an Echinocandin: An Analysis of United States Hospitals
Source: Open Forum Infect Dis. 2024 Jan 3;11(1):ofad703. doi: 10.1093/ofid/ofad703 (PMC10783265; doi:10.1093/ofid/ofad703)
Supplement: ofad703_Supplementary_Data [file ofad703_supplementary_data.docx]

**Supplemental Figure 1.** Comparison of Mean Healthcare Cost Post Index Culture Day by Timing of Echinocandin (Empiric, Early Targeted, Late Targeted, and Delayed) Between Patients with Candidemia and Invasive Candidiasis without Candidemia


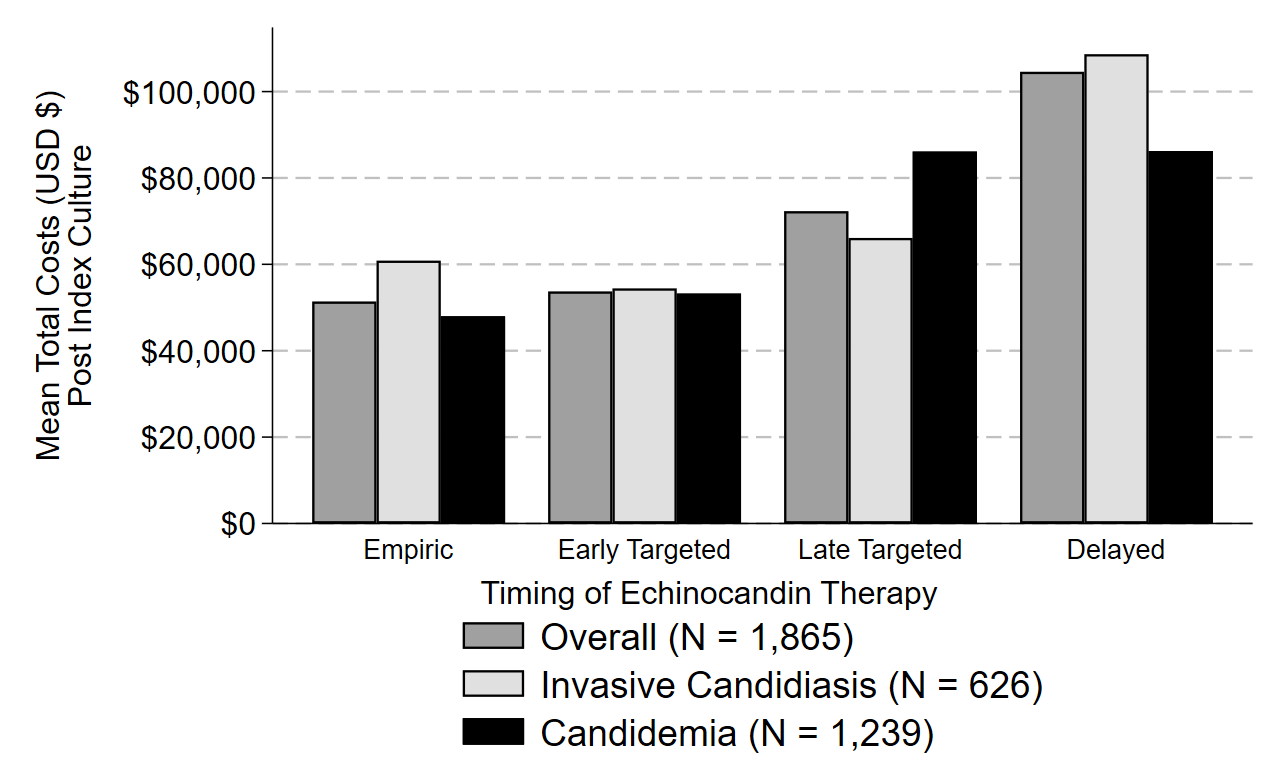


**Supplemental Table 1.** Study Attrition Table

| **Inclusion/Exclusion Criteria** | **Sample Size** |
| --- | --- |
| **Patient admitted on 1/2016 to 12/2019** | 10,656,131 |
| **Patients ≥ 18 years** | 10,351,085 |
| **Patient is an Inpatient** | 2,216,180 |
| ***Candida sp.* on a clinical culture consistent with documented C/IC** | 4,340 |
| **Patient had at least 1 day of echinocandin starting on or after -2 days from the index date** | 2,563 |
| **Three or more consecutive days of echinocandin starting on or after -2 days from index C/IC culture collection day** | 1,916 |
| **First C/IC admission among patients with multiple inpatient admissions with a documented C/IC** | 1,865 |
| **Discharged alive** | 1,599 |
| **Received an echinocandin within day 2 of hospital discharge** | 1,008 |

Abbreviations: C: candidemia; IC: invasive candidiasis without candidemia

**Supplemental Table 2.** List of Culture Sites for the 626 Patients with Invasive Candidiasis

source | Freq. Percent Cum.

----------------------------------------+-----------------------------------

body fluid sample body fluid, other | 268 31.02 31.02

peritoneal fluid sample peritoneal fl.. | 79 9.14 40.16

specimen from abscess abscess drainage | 75 8.68 48.84

tissue specimen soft tissue | 68 7.87 56.71

pleural fluid specimen pleural fluid | 31 3.59 60.30

abdomen | 21 2.43 62.73

bone specimen bone | 21 2.43 65.16

abscess | 17 1.97 67.13

bile specimen biliary tract | 16 1.85 68.98

body fluid sample biliary tract | 13 1.50 70.49

abscess drainage | 12 1.39 71.88

pelvis | 12 1.39 73.26

body fluid specimen body fluid, other | 11 1.27 74.54

peritoneal fluid | 10 1.16 75.69

tissue | 10 1.16 76.85

abdominal fluid | 9 1.04 77.89

peritoneal fluid specimen peritoneal .. | 9 1.04 78.94

specimen obtained by surgical procedu.. | 9 1.04 79.98

knee fluid | 8 0.93 80.90

specimen obtained by aspiration body .. | 8 0.93 81.83

drain jackson-pratt | 7 0.81 82.64

synovial fluid specimen synovial fluid | 7 0.81 83.45

bile | 6 0.69 84.14

fluid (other) | 6 0.69 84.84

specimen obtained by fine needle aspi.. | 6 0.69 85.53

body fluid sample peritoneal fluid | 5 0.58 86.11

drainage fluid sample body fluid, other | 4 0.46 86.57

graft | 4 0.46 87.04

non-biological fluid specimen body fl.. | 4 0.46 87.50

pericardial fluid specimen pericardia.. | 4 0.46 87.96

peritoneal unspecified | 4 0.46 88.43

pleural fluid | 4 0.46 88.89

specimen from abdominal cavity other | 4 0.46 89.35

sternum | 4 0.46 89.81

tissue specimen from lung respiratory.. | 4 0.46 90.28

wound | 4 0.46 90.74

ascites | 3 0.35 91.09

ascitic fluid sample peritoneal fluid | 3 0.35 91.44

biliary fluid | 3 0.35 91.78

cerebrospinal fluid sample cerebrospi.. | 3 0.35 92.13

gastrointestinal tract, other | 3 0.35 92.48

hip | 3 0.35 92.82

pleural fluid pleural fluid | 3 0.35 93.17

tissue specimen skin | 3 0.35 93.52

body fluid specimen skin | 2 0.23 93.75

body fluid, other | 2 0.23 93.98

bone | 2 0.23 94.21

drain unspecified | 2 0.23 94.44

drainage | 2 0.23 94.68

epidural | 2 0.23 94.91

liver | 2 0.23 95.14

specimen from bone bone | 2 0.23 95.37

specimen from liver biliary tract | 2 0.23 95.60

specimen from pancreas bone | 2 0.23 95.83

specimen from unspecified body site o.. | 2 0.23 96.06

specimen from wound abscess drainage | 2 0.23 96.30

specimen obtained by fine needle aspi.. | 2 0.23 96.53

tissue specimen bone | 2 0.23 96.76

tissue specimen groin | 2 0.23 96.99

tricuspid valve | 2 0.23 97.22

biopsy sample other | 1 0.12 97.34

body fluid sample central nervous sys.. | 1 0.12 97.45

body fluid sample wound | 1 0.12 97.57

body fluid specimen biliary tract | 1 0.12 97.69

bone structure bone | 1 0.12 97.80

pancreas | 1 0.12 97.92

pancreatic fluid specimen body fluid,.. | 1 0.12 98.03

paracentesis fluid | 1 0.12 98.15

peritoneal dialysis fluid specimen ot.. | 1 0.12 98.26

peritoneal fluid sample biliary tract | 1 0.12 98.38

sinus fluid sample sinus | 1 0.12 98.50

specimen from central nervous system .. | 1 0.12 98.61

specimen from gallbladder skin | 1 0.12 98.73

specimen from pancreas gastrointestin.. | 1 0.12 98.84

specimen from unspecified body site c.. | 1 0.12 98.96

specimen from unspecified body site o.. | 1 0.12 99.07

specimen from unspecified body site p.. | 1 0.12 99.19

specimen from unspecified body site s.. | 1 0.12 99.31

specimen obtained by aspiration gastr.. | 1 0.12 99.42

specimen obtained by fine needle aspi.. | 1 0.12 99.54

specimen obtained by fine needle aspi.. | 1 0.12 99.65

specimen of unknown material gastroin.. | 1 0.12 99.77

tissue specimen other | 1 0.12 99.88

tissue specimen peritoneal cavity str.. | 1 0.12 100.00

----------------------------------------+-----------------------------------

Total | 864 100.00

There were 864 culture sites among the 626 patients with invasive candidiasis

**Supplemental Table 3:** Select Adjusted Marginal (Mean) Effects on Total Patient Costs from Index Day to Discharge

| **Variable** | **Adjusted Marginal Effect of Total Patient Costs from Index Day to Discharge** | **Mean Difference; 95% Confidence Interval** |
| --- | --- | --- |
| **Timing of Treatment** |  |  |
| Empiric | $51,241 | - |
| Early Targeted | $53,772 | $2,531; 95% CI (-$2,070, $7132) |
| Late Targeted | $66,344 | $15,103; 95% CI ($1,508, $28,698) |
| Delayed | $94,475 | $43,235; 95% CI ($18,792, $67,677) |
| **Age ≥ 70** |  |  |
| No | $57,075 |  |
| Yes | $45,854 | -$11,221; 95% CI (-$15,798, -$6,645) |
| **Hospital Size** |  |  |
| <300 | $47,696 | - |
| 300 to 499 | $43,614 | -$4,081; 95% CI (-$9,636, $1,473) |
| 500+ | $60,270 | $12,574; 95% CI ($7,048, $18,099) |
| **Hospitalization in 6 months prior to index admission** |  |  |
| No | $57,229 |  |
| Yes | $48,897 | -$8,332; 95% CI (-$12,581, -$4,083) |
| **Any Chronic kidney disease diagnosis at admission** |  |  |
| No | $51,424 |  |
| Yes | $62,658 | $11,234; 95% CI ($5,154, $17,314) |
| **Cardiac Arrhythmia** |  |  |
| No | $50,442 |  |
| Yes | $57,816 | $7,374; 95% CI ($2,665, $12,083) |
| **Pulmonary circulation disease** |  |  |
| No | $51,512 |  |
| Yes | $65,766 | $14,254; 95% CI ($6,610, $21,898) |
| **Coagulopathy** |  |  |
| No | $49,646 |  |
| Yes | $61,051 | $11,405; 95% CI ($6,104, $16,707) |
| **Weight loss** |  |  |
| No | $47,847 |  |
| Yes | $60,738 | $12,891; 95% CI ($8,411, $17,371) |
| **Depression** |  |  |
| No | $50,668 |  |
| Yes | $63,317 | $12,649; 95% CI ($6,991, $18,308) |
| **Mechanical Ventilation on Index Day** |  |  |
| No | $47,045 |  |
| Yes | $64,763 | $17,718; 95% CI ($10,704, $24,732) |
| **ICU Residence on Index Day** |  |  |
| No | $48,878 |  |
| Yes | $57,517 | $8,639; 95%CI ($2,897, $14,381) |
| **Treatment received between admission and index C/IC day** |  |  |
| **Fluconazole** |  |  |
| No | $52,280 |  |
| Yes | $59,857 | $7,577; 95% CI ($1,329, $13,826) |
| **Total Parenteral Nutrition** |  |  |
| No | $52,431 |  |
| Yes | $68,423 | $15,991; 95% CI ($5,700, $26,282) |
| **Vasopressors** |  |  |
| No | $49,992 |  |
| Yes | $56,513 | $6,521; 95% CI ($1,381, $11,663) |
| **Diuretics** |  |  |
| No | $50,968 |  |
| Yes | $58,558 | $7,590; 95% CI ($2,402, $12,777) |
| **Inotropes** |  |  |
| No | $52,428 |  |
| Yes | $72,004 | $19,576; 95% CI ($4,833, $34,319) |
| **Died in-Hospital** |  |  |
| No | $52,472 |  |
| Yes | $59,260 | $6,788; 95% CI (-$140, $13,717) |

**Supplemental Table 4.** Comparison of Baseline Characteristics and Clinical Covariates between Patients Who Were Discharged Alive and Were Receiving an Echinocandin Within 2 Days of Hospital Discharge

| **Baseline Characteristics** | **Discharged Alive**  **N = 1599** | **%** | **Received an echinocandin within day 2 of Hospital Discharge**  **N = 1,008** | **%** |
| --- | --- | --- | --- | --- |
| **Hospital Characteristics** | | | | |
| **Census region** |  |  |  |  |
| Midwest | 385 | 24.08% | 254 | 25.20% |
| Northeast | 146 | 9.13% | 87 | 8.63% |
| South | 1067 | 66.73% | 667 | 66.17% |
| West | 1 | 0.06% | 0 | 0.00% |
| **Number of beds** |  |  |  |  |
| <300 | 289 | 18.07% | 186 | 18.45% |
| 300 to 499 | 427 | 26.70% | 299 | 29.66% |
| 500+ | 883 | 55.22% | 523 | 51.88% |
| **Teaching vs non-teaching hospital** | 1030 | 64.42% | 632 | 62.70% |
| **Urban vs rural location of hospital** | 1452 | 90.81% | 912 | 90.48% |
| **Demographics** | | | | |
| **Mean (SD) age, years** | 58.3 (16.1) |  | 58.8 (16.1) |  |
| **Sex: Female** | 785 | 49.09% | 488 | 48.41% |
| **Race** |  |  |  |  |
| White | 1236 | 77.30% | 789 | 78.27% |
| Black | 232 | 14.51% | 136 | 13.49% |
| Asian | 9 | 0.56% | 8 | 0.79% |
| Other | 85 | 5.32% | 52 | 5.16% |
| Unknown | 37 | 2.31% | 23 | 2.28% |
| **Hispanic Ethnicity** | 55 | 3.44% | 30 | 2.98% |
| **Admission Source** |  |  |  |  |
| Non-healthcare facility (including home) | 1094 | 68.42% | 700 | 69.44% |
| Clinic | 112 | 7.00% | 83 | 8.23% |
| Transfer from Another Hospital | 326 | 20.39% | 178 | 17.66% |
| Transfer from SNF, ICF | 34 | 2.13% | 23 | 2.28% |
| Other | 33 | 2.06% | 24 | 2.38% |
| Payer |  |  |  |  |
| Medicare | 855 | 53.47% | 548 | 54.37% |
| Medicaid | 323 | 20.20% | 191 | 18.95% |
| Managed Care | 239 | 14.95% | 168 | 16.67% |
| Commercial | 66 | 4.13% | 37 | 3.67% |
| Other | 116 | 7.25% | 64 | 6.35% |
| **Medical History and Comorbidities** | | | | |
| **Hospitalization in 6 months prior to index admission** | 715 | 44.72% | 484 | 48.02% |
| **Any Chronic kidney disease diagnosis at admission** | 295 | 18.45% | 187 | 18.55% |
| **Elixhauser Comorbidities** |  |  |  |  |
| Congestive heart failure | 430 | 26.89% | 250 | 24.80% |
| Cardiac Arrhythmia | 591 | 36.96% | 360 | 35.71% |
| Valvular disease | 214 | 13.38% | 128 | 12.70% |
| Pulmonary circulation disease | 198 | 12.38% | 109 | 10.81% |
| Peripheral vascular disease | 193 | 12.07% | 131 | 13.00% |
| Paralysis | 65 | 4.07% | 31 | 3.08% |
| Other neurological disorders | 465 | 29.08% | 247 | 24.50% |
| Chronic pulmonary disease | 455 | 28.46% | 283 | 28.08% |
| Diabetes without chronic complications | 156 | 9.76% | 101 | 10.02% |
| Diabetes with chronic complications | 463 | 28.96% | 286 | 28.37% |
| Hypothyroidism | 222 | 13.88% | 136 | 13.49% |
| Renal failure | 474 | 29.64% | 292 | 28.97% |
| Liver disease | 262 | 16.39% | 151 | 14.98% |
| Peptic ulcer disease with bleeding | 37 | 2.31% | 23 | 2.28% |
| AIDS | 15 | 0.94% | 9 | 0.89% |
| Lymphoma | 28 | 1.75% | 19 | 1.88% |
| Metastatic cancer | 126 | 7.88% | 82 | 8.13% |
| Solid tumor without metastasis | 210 | 13.13% | 133 | 13.19% |
| Rheumatoid arthritis/collagen vascular | 78 | 4.88% | 43 | 4.27% |
| Coagulopathy | 388 | 24.27% | 217 | 21.53% |
| Obesity | 337 | 21.08% | 205 | 20.34% |
| Weight loss | 665 | 41.59% | 375 | 37.20% |
| Fluid and electrolyte disorders | 1160 | 72.55% | 691 | 68.55% |
| Chronic blood loss anemia | 35 | 2.19% | 19 | 1.88% |
| Deficiency anemia | 181 | 11.32% | 106 | 10.52% |
| Alcohol abuse | 148 | 9.26% | 87 | 8.63% |
| Drug abuse | 266 | 16.64% | 165 | 16.37% |
| Psychosis | 45 | 2.81% | 23 | 2.28% |
| Depression | 387 | 24.20% | 222 | 22.02% |
| Hypertension | 1023 | 63.98% | 647 | 64.19% |
| **Charlson Comorbidities** |  |  |  |  |
| Acute Myocardial Infarction | 169 | 10.57% | 108 | 10.71% |
| CHF | 430 | 26.89% | 250 | 24.80% |
| Peripheral vascular disease | 193 | 12.07% | 131 | 13.00% |
| Cerebrovascular Disease | 141 | 8.82% | 83 | 8.23% |
| Dementia | 81 | 5.07% | 54 | 5.36% |
| COPD | 455 | 28.46% | 283 | 28.08% |
| Rheumatoid Disease | 66 | 4.13% | 38 | 3.77% |
| Peptic Ulcer Disease | 133 | 8.32% | 76 | 7.54% |
| Mild Liver Disease | 135 | 8.44% | 78 | 7.74% |
| Diabetes | 287 | 17.95% | 180 | 17.86% |
| Diabetes with Complications | 332 | 20.76% | 207 | 20.54% |
| Hemiplegia or Paraplegia | 65 | 4.07% | 31 | 3.08% |
| Renal Disease | 474 | 29.64% | 292 | 28.97% |
| Cancer | 159 | 9.94% | 98 | 9.72% |
| Moderate/Severe Liver Disease | 72 | 4.50% | 46 | 4.56% |
| Metastatic cancer | 126 | 7.88% | 82 | 8.13% |
| AIDS | 15 | 0.94% | 9 | 0.89% |
| **Charlson Comorbidity Score** |  |  |  |  |
| 0 | 244 | 15.26% | 164 | 16.27% |
| 1 | 237 | 14.82% | 148 | 14.68% |
| 2 | 261 | 16.32% | 163 | 16.17% |
| 3 | 212 | 13.26% | 131 | 13.00% |
| 4 | 174 | 10.88% | 110 | 10.91% |
| 5+ | 471 | 29.46% | 292 | 28.97% |
| Mean (SD) | 3.3 (2.6) |  | 3.2 (2.6) |  |
| **Hospitalization Course** | | | | |
| **LOS prior to index C/IC culture** |  |  |  |  |
| Mean (SD) | 7.1 (10.0) |  | 5.7 (7.7) |  |
| Median (IQR) | 4 [1, 10] |  | 2 [1, 8] |  |
| **Residence in ICU on index C/IC culture day** | 639 | 39.96% | 357 | 35.42% |
| **Days in ICU prior to index C/IC culture day for patients in the ICU prior to index** | 6.5 (8.0); n = 521 |  | 6.0 (7.0); n = 277 |  |
| **Mechanical ventilation on index C/IC culture day** | 363 | 22.70% | 187 | 18.55% |
| **Days on mechanical ventilation prior to index C/IC culture day for patients on MV prior to index** | 6.6 (8.3); N = 343 |  | 5.4 (6.1); N = 163 |  |
| **Microbiology, Infection, and Treatment Characteristics** | | | | |
| **Infection** **type** |  |  |  |  |
| C | 1007 | 62.98% | 624 | 61.90% |
| IC | 560 | 35.02% | 361 | 35.81% |
| C and IC | 32 | 2.00% | 24 | 2.38% |
| ***Candida sp.* ± 3 days of index C/IC culture** |  |  |  |  |
| *C. albicans* | 587 | 36.71% | 317 | 31.45% |
| *C. glabrata* | 459 | 28.71% | 342 | 33.93% |
| *C. parapsilosis* | 196 | 12.26% | 125 | 12.40% |
| *C. tropicalis* | 159 | 9.94% | 101 | 10.02% |
| *Other* | 303 | 18.95% | 191 | 18.95% |
| **Number of *Candida sp.* ± 3 days of index C/IC culture** |  |  |  |  |
| 1 | 1498 | 93.68% | 943 | 93.55% |
| 2 | 97 | 6.07% | 62 | 6.15% |
| 3+ | 4 | 0.25% | 3 | 0.30% |
| **Antifungals received between admission and index C/IC day** |  |  |  |  |
| Fluconazole | 269 | 16.82% | 160 | 15.87% |
| Voriconazole | 4 | 0.25% | 2 | 0.20% |
| Posaconazole | 5 | 0.31% | 2 | 0.20% |
| Isavuconazole | 3 | 0.19% | 1 | 0.10% |
| Anidulafungin | 5 | 0.31% | 5 | 0.50% |
| Micafungin | 176 | 11.01% | 97 | 9.62% |
| Caspofungin | 33 | 2.06% | 14 | 1.39% |
| **Number of antifungals received between admission and index C/IC day** |  |  |  |  |
| 0 | 1142 | 71.42% | 748 | 74.21% |
| 1 | 419 | 26.20% | 239 | 23.71% |
| 2 | 38 | 2.38% | 21 | 2.08% |
| **Antibiotics received between admission and index C/IC day** |  |  |  |  |
| Aminoglycosides | 113 | 7.07% | 59 | 5.85% |
| Β-lactam | 1454 | 90.93% | 913 | 90.58% |
| Fluoroquinolone | 245 | 15.32% | 154 | 15.28% |
| Vancomycin | 985 | 61.60% | 580 | 57.54% |
| Daptomycin | 73 | 4.57% | 42 | 4.17% |
| Macrolide | 143 | 8.94% | 82 | 8.13% |
| Oxazolidones | 98 | 6.13% | 52 | 5.16% |
| Polymyxins | 9 | 0.56% | 4 | 0.40% |
| Rifamycin | 1 | 0.06% | 0 | 0.00% |
| Sulfa | 28 | 1.75% | 13 | 1.29% |
| Tetracycline | 63 | 3.94% | 37 | 3.67% |
| Other (fosfomycin, nitrofurantoin) | 4 | 0.25% | 0 | 0.00% |
| **Number of antibiotics received between admission and index treatment day** |  |  |  |  |
| 0 | 78 | 4.88% | 54 | 5.36% |
| 1 | 403 | 25.20% | 282 | 27.98% |
| 2 | 690 | 43.15% | 433 | 42.96% |
| ≥3 | 428 | 26.77% | 239 | 23.71% |
| **Echinocandin treatment initiation relative to index C/IC Day** |  |  |  |  |
| -2-2 days of C/IC | 998 | 62.41% | 607 | 60.22% |
| 3-6 days post C/IC | 506 | 31.64% | 335 | 33.23% |
| 7-10 days post C/IC | 62 | 3.88% | 43 | 4.27% |
| 11+ days post C/IC | 33 | 2.06% | 23 | 2.28% |
| **Duration of echinocandin treatment** |  |  |  |  |
| Mean (SD) | 9.3 (8.0) |  | 9.5 (7.8) |  |
| Median (IQR) | 7 [4, 12] |  | 7 [5, 12] |  |
| **Other antifungals received from index echinocandin treatment day through discharge** |  |  |  |  |
| Fluconazole | 827 | 51.72% | 393 | 38.99% |
| Voriconazole | 66 | 4.13% | 40 | 3.97% |
| Posaconazole | 4 | 0.25% | 1 | 0.10% |
| Anti-fungal therapies administered concurrently with the echinocandin treatment |  |  |  |  |
| Fluconazole | 483 | 30.21% | 288 | 28.57% |
| Voriconazole | 39 | 2.44% | 26 | 2.58% |
| Posaconazole | 4 | 0.25% | 1 | 0.10% |
| **Anti-fungal therapies administered post-discontinuation of echinocandin therapy** |  |  |  |  |
| Fluconazole | 53 | 3.31% | 186 | 18.45% |
| Voriconazole | 43 | 2.69% | 22 | 2.18% |
| Posaconazole | 2 | 0.13% | 1 | 0.10% |

Abbreviations: SD: standard deviation; SNF: skilled nursing facility; ICF: intermediate care facility; CHF: congestive heart failure; COPD: chronic obstructive pulmonary disease; AIDs: acquired immunodeficiency syndrome; LOS: length of stay; C/IC: C: candidemia; IC: invasive candidiasis without candidemia; IQR: interquartile range; ICU: intensive care unit; MV: mechanical ventilation.

**Supplemental Table 5.** Comparison of Patients Who Were Receiving an Echinocandin Near Hospital Discharge and Met the Criteria for a Potentially Earlier Hospital Discharge Relative to Patients Who Were Receiving an Echinocandin Near Hospital Discharge and Did Not Meet the Potentially Earlier Hospital Discharge Criteria

| **Baseline Characteristics** | **Eligible for Early Discharge** | | **%** | **Not Eligible for Early Discharge** | | **%** |  |
| --- | --- | --- | --- | --- | --- | --- | --- |
|  | **N = 432** | |  | **N = 576** | |  | P-value |
| **Hospital Characteristics** | | | | | | |  |
| **Census region** |  |  | |  |  | |  |
| Midwest | 102 | 23.61% | | 152 | 26.39% | |  |
| Northeast | 39 | 9.03% | | 48 | 8.33% | | 0.69 |
| South | 291 | 67.36% | | 376 | 65.28% | |  |
| West | 0 | 0.00% | | 0 | 0.00% | |  |
| **Number of beds** |  |  | |  |  | |  |
| <300 | 74 | 17.13% | | 112 | 19.44% | |  |
| 300 to 499 | 125 | 28.94% | | 174 | 30.21% | | 0.48 |
| 500+ | 233 | 53.94% | | 290 | 50.35% | |  |
| **Teaching vs non-teaching hospital** | 278 | 64.35% | | 354 | 61.46% | | 0.35 |
| **Urban vs rural location of hospital** | 398 | 92.13% | | 514 | 89.24% | | 0.12 |
| **Demographics** | | | | | | |  |
| **Mean (SD) age, years** | 57.4 (16.3) |  | | 59.9 (15.9) |  | | 0.01 |
| **Sex: Female** | 206 | 47.69% | | 304 | 52.78% | | 0.69 |
| **Race** |  |  | |  |  | |  |
| White | 333 | 77.08% | | 456 | 79.17% | |  |
| Black | 64 | 14.81% | | 72 | 12.50% | |  |
| Asian | 5 | 1.16% | | 3 | 0.52% | | 0.26 |
| Other | 24 | 5.56% | | 28 | 4.86% | |  |
| Unknown | 6 | 1.39% | | 17 | 2.95% | |  |
| **Hispanic Ethnicity** | 13 | 3.01% | | 17 | 2.95% | | 0.96 |
| **Admission Source** |  |  | |  |  | |  |
| Non-healthcare facility (including from home) | 310 | 71.76% | | 390 | 67.71% | |  |
| Clinic | 35 | 8.10% | | 48 | 8.33% | |  |
| Transfer from Another Hospital | 65 | 15.05% | | 113 | 19.62% | | 0.07 |
| Transfer from SNF, ICF | 7 | 1.62% | | 16 | 2.78% | |  |
| Other | 15 | 3.47% | | 9 | 1.56% | |  |
| Payer |  |  | |  |  | |  |
| Medicare | 219 | 50.69% | | 329 | 57.12% | |  |
| Medicaid | 96 | 22.22% | | 95 | 16.49% | |  |
| Managed Care | 70 | 16.20% | | 98 | 17.01% | | 0.04 |
| Commercial | 13 | 3.01% | | 24 | 4.17% | |  |
| Other | 34 | 7.87% | | 30 | 5.21% | |  |
| **Medical History and Comorbidities** | | | | | | |  |
| **Hospitalization in 6 months prior to index admission** | 215 | 49.77% | | 269 | 46.70% | | 0.34 |
| **Any Chronic kidney disease diagnosis at admission** | 70 | 16.20% | | 117 | 20.31% | | 0.10 |
| **Elixhauser Comorbidities** |  |  | |  | 0.00% | |  |
| Congestive heart failure | 86 | 19.91% | | 164 | 28.47% | | <0.01 |
| Cardiac Arrhythmia | 127 | 29.40% | | 233 | 40.45% | | <0.01 |
| Valvular disease | 40 | 9.26% | | 88 | 15.28% | | <0.01 |
| Pulmonary circulation disease | 40 | 9.26% | | 69 | 11.98% | | 0.17 |
| Peripheral vascular disease | 54 | 12.50% | | 77 | 13.37% | | 0.69 |
| Paralysis | 17 | 3.94% | | 14 | 2.43% | | 0.17 |
| Other neurological disorders | 88 | 20.37% | | 159 | 27.60% | | <0.01 |
| Chronic pulmonary disease | 112 | 25.93% | | 171 | 29.69% | | 0.19 |
| Diabetes without chronic complications | 41 | 9.49% | | 60 | 10.42% | | 0.63 |
| Diabetes with chronic complications | 123 | 28.47% | | 163 | 28.30% | | 0.95 |
| Hypothyroidism | 63 | 14.58% | | 73 | 12.67% | | 0.38 |
| Renal failure | 106 | 24.54% | | 186 | 32.29% | | <0.01 |
| Liver disease | 57 | 13.19% | | 94 | 16.32% | | 0.17 |
| Peptic ulcer disease with bleeding | 7 | 1.62% | | 16 | 2.78% | | 0.22 |
| AIDS | 5 | 1.16% | | 4 | 0.69% | | 0.51 |
| Lymphoma | 9 | 2.08% | | 10 | 1.74% | | 0.69 |
| Metastatic cancer | 32 | 7.41% | | 50 | 8.68% | | 0.46 |
| Solid tumor without metastasis | 55 | 12.73% | | 78 | 13.54% | | 0.71 |
| Rheumatoid arthritis/collagen vascular | 18 | 4.17% | | 25 | 4.34% | | 0.89 |
| Coagulopathy | 70 | 16.20% | | 147 | 25.52% | | <0.01 |
| Obesity | 86 | 19.91% | | 119 | 20.66% | | 0.77 |
| Weight loss | 147 | 34.03% | | 228 | 39.58% | | 0.07 |
| Fluid and electrolyte disorders | 282 | 65.28% | | 409 | 71.01% | | 0.05 |
| Chronic blood loss anemia | 8 | 1.85% | | 11 | 1.91% | | 0.95 |
| Deficiency anemia | 50 | 11.57% | | 56 | 9.72% | | 0.34 |
| Alcohol abuse | 39 | 9.03% | | 48 | 8.33% | | 0.70 |
| Drug abuse | 88 | 20.37% | | 77 | 13.37% | | <0.01 |
| Psychosis | 11 | 2.55% | | 12 | 2.08% | | 0.63 |
| Depression | 103 | 23.84% | | 119 | 20.66% | | 0.23 |
| Hypertension | 273 | 63.19% | | 374 | 64.93% | | 0.57 |
| **Charlson Comorbidities** |  |  | |  |  | |  |
| Acute Myocardial Infarction | 48 | 11.11% | | 60 | 10.42% | | 0.72 |
| CHF | 86 | 19.91% | | 164 | 28.47% | | <0.01 |
| Peripheral vascular disease | 54 | 12.50% | | 77 | 13.37% | | 0.69 |
| Cerebrovascular Disease | 29 | 6.71% | | 54 | 9.38% | | 0.13 |
| Dementia | 19 | 4.40% | | 35 | 6.08% | | 0.24 |
| COPD | 112 | 25.93% | | 171 | 29.69% | | 0.19 |
| Rheumatoid Disease | 16 | 3.70% | | 22 | 3.82% | | 0.92 |
| Peptic Ulcer Disease | 26 | 6.02% | | 50 | 8.68% | | 0.11 |
| Mild Liver Disease | 38 | 8.80% | | 40 | 6.94% | | 0.28 |
| Diabetes | 78 | 18.06% | | 102 | 17.71% | | 0.89 |
| Diabetes with Complications | 86 | 19.91% | | 121 | 21.01% | | 0.67 |
| Hemiplegia or Paraplegia | 17 | 3.94% | | 14 | 2.43% | | 0.17 |
| Renal Disease | 106 | 24.54% | | 186 | 32.29% | | <0.01 |
| Cancer | 42 | 9.72% | | 56 | 9.72% | | 1.000 |
| Moderate/Severe Liver Disease | 12 | 2.78% | | 34 | 5.90% | | <0.01 |
| Metastatic cancer | 32 | 7.41% | | 50 | 8.68% | | 0.46 |
| AIDS | 5 | 1.16% | | 4 | 0.69% | | 0.51 |
| **Charlson Comorbidity Score** |  |  | |  |  | |  |
| 0 | 83 | 19.21% | | 81 | 14.06% | |  |
| 1 | 66 | 15.28% | | 82 | 14.24% | |  |
| 2 | 72 | 16.67% | | 91 | 15.80% | |  |
| 3 | 65 | 15.05% | | 66 | 11.46% | | 0.01 |
| 4 | 36 | 8.33% | | 74 | 12.85% | |  |
| 5+ | 110 | 25.46% | | 182 | 31.60% | |  |
| Mean (SD) | 2.9 (2.6) |  | | 3.4 (2.6) |  | | <0.01 |
| **Hospitalization Course** | | | | | | |  |
| **LOS prior to index C/IC culture** |  |  | |  |  | |  |
| Mean (SD) | 5.5 (8.0) |  | | 5.9 (7.5) |  | | 0.37 |
| Median (IQR) | 2 [1, 6] |  | | 2 [1, 8] |  | | 0.07 |
| **Residence in ICU on index C/IC culture day** | 123 | 28.47% | | 234 | 40.63% | | <0.01 |
| **Mean (SD) days in ICU prior to index C/IC culture day for patients in the ICU prior to index** | 3.4 (3.4); n =79 |  | | 6.1 (6.7); n = 198 |  | | <0.01 |
| **Mechanical ventilation on index C/IC culture day** | 51 | 11.81% | | 136 | 23.61% | | <0.01 |
| **Mean (SD) days on mechanical ventilation prior to index C/IC culture day for patients on MV prior to index** | 3.6 (2.6); N = 34 |  | | 5.9 (6.6); N = 129 |  | | 0.06 |
| **Microbiology, Infection, and Treatment Characteristics** | | | | | | |  |
| **Infection type** |  |  | |  |  | |  |
| C | 271 | 62.73% | | 353 | 61.28% | |  |
| IC | 150 | 34.72% | | 211 | 36.63% | | 0.75 |
| C and IC | 11 | 2.55% | | 12 | 2.08% | |  |
| ***Candida sp.* ± 3 days of index C/IC culture** |  |  | |  |  | |  |
| *C. albicans* | 133 | 30.79% | | 184 | 31.94% | | 0.70 |
| *C. glabrata* | 148 | 34.26% | | 194 | 33.68% | | 0.85 |
| *C. parapsilosis* | 51 | 11.81% | | 74 | 12.85% | | 0.62 |
| *C. tropicalis* | 43 | 9.95% | | 58 | 10.07% | | 0.95 |
| *Other* | 84 | 19.44% | | 107 | 18.58% | | 0.73 |
| **Number of *Candida sp.* ± 3 days of index C/IC culture** |  |  | |  |  | |  |
| 1 | 406 | 93.98% | | 537 | 93.23% | |  |
| 2 | 25 | 5.79% | | 37 | 6.42% | | 0.87 |
| 3+ | 1 | 0.23% | | 2 | 0.35% | |  |
| **Antifungals received between admission and index C/IC day** |  |  | |  |  | |  |
| Fluconazole | 52 | 12.04% | | 108 | 18.75% | | <0.01 |
| Voriconazole | 2 | 0.46% | | 0 | 0.00% | | 0.18 |
| Posaconazole | 0 | 0.00% | | 2 | 0.35% | | 0.51 |
| Isavuconazole | 0 | 0.00% | | 1 | 0.17% | | 1.000 |
| Anidulafungin | 2 | 0.46% | | 3 | 0.52% | | 1.000 |
| Micafungin | 33 | 7.64% | | 64 | 11.11% | | 0.06 |
| Caspofungin | 4 | 0.93% | | 10 | 1.74% | | 0.42 |
| **Number of antifungals received between admission and index C/IC day** |  |  | |  |  | |  |
| 0 | 344 | 79.63% | | 404 | 70.14% | |  |
| 1 | 83 | 19.21% | | 156 | 27.08% | | <0.01 |
| 2 | 5 | 1.16% | | 16 | 2.78% | |  |
| **Antibiotics received between admission and index C/IC day** |  |  | |  |  | |  |
| Aminoglycosides | 25 | 5.79% | | 34 | 5.90% | | 0.94 |
| Β-lactam | 391 | 90.51% | | 522 | 90.63% | | 0.95 |
| Fluoroquinolone | 55 | 12.73% | | 99 | 17.19% | | 0.05 |
| Vancomycin | 235 | 54.40% | | 345 | 59.90% | | 0.08 |
| Daptomycin | 13 | 3.01% | | 29 | 5.03% | | 0.11 |
| Macrolide | 27 | 6.25% | | 55 | 9.55% | | 0.06 |
| Oxazolidones | 11 | 2.55% | | 41 | 7.12% | | <0.01 |
| Polymyxins | 2 | 0.46% | | 2 | 0.35% | | 1.000 |
| Rifamycin | 0 | 0.00% | | 0 | 0.00% | | 1.000 |
| Sulfa | 4 | 0.93% | | 9 | 1.56% | | 0.42 |
| Tetracycline | 15 | 3.47% | | 22 | 3.82% | | 0.77 |
| Other (fosfomycin, nitrofurantoin) | 1 | 0.23% | | 2 | 0.35% | | 1.000 |
| **Number of antibiotics received between admission and index treatment day** |  |  | |  |  | |  |
| 0 | 25 | 5.79% | | 29 | 5.03% | |  |
| 1 | 142 | 32.87% | | 140 | 24.31% | | <0.01 |
| 2 | 182 | 42.13% | | 251 | 43.58% | |  |
| ≥3 | 83 | 19.21% | | 156 | 27.08% | |  |
| **Echinocandin treatment initiation relative to index C/IC day** |  |  | |  |  | |  |
| -2-2 days of C/IC | 258 | 59.72% | | 349 | 60.59% | |  |
| 3-6 days post C/IC | 149 | 34.49% | | 186 | 32.29% | | 0.68 |
| 7-10 days post C/IC | 15 | 3.47% | | 28 | 4.86% | |  |
| 11+ days post C/IC | 10 | 2.31% | | 13 | 2.26% | |  |
| **Duration of echinocandin treatment** |  |  | |  |  | |  |
| Mean (SD) | 10.1 (9.5) |  | | 8.9 (6.2) |  | | 0.02 |
| Median (IQR) | 7 [5, 13] |  | | 7 [5, 12] |  | | 0.31 |
| **Other antifungals received from index echinocandin treatment day through discharge** |  |  | |  |  | |  |
| Fluconazole | 186 | 42.53% | | 207 | 36.77% | | 0.02 |
| Voriconazole | 24 | 5.67% | | 16 | 2.90% | | 0.03 |
| Posaconazole | 0 | 0.00% | | 1 | 0.16% | | 1.000 |
| **Anti-fungal therapies administered concurrently with the echinocandin treatment** |  |  | |  |  | |  |
| Fluconazole | 125 | 28.09% | | 163 | 28.87% | | 0.83 |
| Voriconazole | 15 | 3.61% | | 11 | 1.94% | | 0.16 |
| Posaconazole | 0 | 0.00% | | 1 | 0.16% | | 1.000 |
| **Anti-fungal therapies administered post-discontinuation of echinocandin therapy** |  |  | |  |  | |  |
| Fluconazole | 95 | 22.42% | | 91 | 15.97% | | 0.01 |
| Voriconazole | 14 | 3.35% | | 8 | 1.45% | | 0.05 |
| Posaconazole | 0 | 0.00% | | 1 | 0.16% | | 1.000 |

Abbreviations: SD: standard deviation; SNF: skilled nursing facility; ICF: intermediate care facility; CHF: congestive heart failure; COPD: chronic obstructive pulmonary disease; AIDs: acquired immunodeficiency syndrome; LOS: length of stay; C/IC: C: candidemia; IC: invasive candidiasis without candidemia; IQR: interquartile range; ICU: intensive care unit; MV: mechanical ventilation.
